# Valve Heart Surgery in Brazil - The BYPASS Registry Analysis

**DOI:** 10.21470/1678-9741-2019-0408

**Published:** 2020

**Authors:** Alexandre Cabral Zilli, Solange Guizilini, Isadora S. Rocco, José Amalth do Espírito Santo, Otavio Berwanger, Renato Abdala Karam Kalil, Fabio Biscegli Jatene, Alexandre Biasi Cavalcanti, Renato Hideo Nakagawa Santos, Walter J. Gomes, BYPASS Registry Study Group

**Affiliations:** 1 Discipline of Cardiovascular Surgery, Hospital São Paulo, Escola Paulista de Medicina, Universidade Federal de São Paulo - EPM-UNIFESP, São Paulo, SP, Brazil.; 2 Instituto de Pesquisa do Hospital do Coração - IP-HCor, São Paulo, SP, Brazil.; 3 Instituto de Cardiologia do Rio Grande do Sul, Fundação Universitária de Cardiologia, Porto Alegre, RS, Brazil.; 4 Cardiovascular Surgery Division, Instituto do Coração do Hospital das Clínicas da Faculdade de Medicina da Universidade de São Paulo - InCor-HCFMUSP, São Paulo, SP, Brazil.

**Keywords:** Aortic Valve, Hospital Mortality, Public Health, Access to Information, Heart Valve Diseases, Cardiac Surgical Procedures

## Abstract

**Objective:**

To analyze the profile and outcomes of patients who underwent valve heart surgery in Brazil, using information retrieved from the Brazilian Registry of Cardiovascular Surgeries in Adults (BYPASS Registry) database.

**Methods:**

This is a multicenter cohort study, evaluating 920 patients submitted to heart valve surgery. Demographics and postoperative clinical outcomes were assessed and compared to estimate mortality risk using the European System for Cardiac Operative Risk Evaluation (EuroSCORE).

**Results:**

Isolated aortic valve replacement was the most frequently performed surgery (34%), followed by isolated mitral valve replacement (24.9%). Valve repair was performed in 21% of mitral procedures. Minimally invasive access was performed in 1.6% and the most frequent postoperative complications were arrhythmias (22.6%), infections (5.7%), and low-output syndrome (5.1%). Operations covered by the public health system accounted for 80.8% and the hospital mortality rate was 7.3%.

**Conclusion:**

The most frequent isolated valve surgery in Brazil is the aortic valve replacement by conventional open access and the rheumatic disease is still the main etiology for valve surgery. The BYPASS Registry has a fundamental role to provide information on the profile of patients with valve heart disease in our country in order to delineate adequate strategies for health promotion and resource allocation for cardiac surgery.

**Table:** 

Abbreviations, acronyms & symbols				
AVR	= Aortic valve replacement		MVR	= Mitral valve replacement
AVR+CABG	= Aortic valve replacement + Coronary artery bypass grafting		MVR+CABG	= Mitral valve replacement + Coronary artery bypass grafting
AVR+MVRr	= Aortic valve replacement + Mitral valve replacement or repair		MVRr+TVr	= Mitral valve replacement or repair + Tricuspid valve repair
BSCVS	= Brazilian Society of Cardiovascular Surgery		NYHA	= New York Heart Association
BYPASS Registry	= Brazilian Registry of Cardiovascular Surgery in Adults		RVD	= Rheumatic valve disease
CABG	= Coronary artery bypass grafting		SAVR	= Surgical aortic valve replacement
COPD	= Chronic obstructive pulmonary disease		SD	= Standard deviation
DATASUS	= Departamento de Informática do SUS (the Brazilian Health Informatics Department)		STS	= Society of Thoracic Surgeons
EACTS	= European Association of Cardiothoracic Surgery		SUS	= Sistema Único de Saúde (the Brazilian National Health System)
EuroSCORE	= European System for Cardiac Operative Risk Evaluation		TAVR	= Transcatheter aortic valve replacement
ICU	= Intensive care unit		TVr	= Tricuspid valve repair
LVEF	= Left ventricular ejection fraction		VHD	= Valve heart diseases
MVr	= Mitral valve repair		VHS	= Valve heart surgery
MVr+CABG	= Mitral valve repair + Coronary artery bypass grafting			

## INTRODUCTION

Valve heart diseases (VHD) represent the second most common indication for cardiac surgery in the world, accounting for nearly one-third of the cardiac operations performed^[1]^. However, its etiology and clinical characteristics differ widely according to the population and country studied. In Brazil, scarce data exists so far on the epidemiological profile, primary payers, and outcomes of the surgical treatment of patients with VHD. Therefore, it is essential to understand the profile of patients with VHD in our country to delineate adequate strategies for health promotion and allocation of resources for cardiac surgery. Following the establishment of the Society of Thoracic Surgeons (STS) Adult Cardiac Surgery Database, in 1989, to address the shortcomings of coronary artery bypass grafting (CABG) mortality data, many other national and continental databases were instituted to gather information on current trends and improve the quality assessment and advance of cardiovascular surgery in their respective areas^[2]^.

An important headway was provided with the inception of the first Brazilian national database of cardiovascular surgery in adults, the BYPASS Registry^[3,4]^. The BYPASS Registry is an ongoing database currently collecting information from 17 participating centers across the country, and steadily expanding the pool of contributing units.

Therefore, the objective of the current study was to analyze the profile and outcomes of patients who underwent valve heart surgery (VHS) in Brazil, using information retrieved from the BYPASS Registry database.

## METHODS

This is a multicenter observational/prospective cohort study using data from the BYPASS Registry database. The BYPASS project is a national heart surgery registry, owned and funded by the Brazilian Society of Cardiovascular Surgery (BSCVS) and coordinated in partnership with the Research Institute of the Hospital do Coração - IP-HCor, in São Paulo, Brazil.

The participation of cardiovascular surgery centers in the BYPASS project was voluntarily convened and involves institutions located across the whole national territory. The 17 participating centers are well distributed among the following regions of the country: Southeast (n=8), Northeast (n=5), South (n=3), and Midwest (n=1). The informed consent form was signed by each patient following the national standards of clinical research already approved by the ethics and research committee of the coordinating center and each participating institution. All participating institutions were requested to complete the structured questionnaire, pertaining to all performed procedures and the related outcomes.

Adult patients over 18 years of age submitted to valve operations (isolated and/or combined) from August 2014 to April 2018 were prospectively included in the current analysis. Patients less than 18 years of age or those who refused to sign the informed consent form were excluded.

### Outcomes

Initially, the patients were analyzed according to the etiopathogenesis of the valve disease: rheumatic valve disease (RVD), congenital, degenerative senile aortic, infectious/endocarditis, degenerative mitral/leaflet prolapse/chordae rupture, ischemic, prosthesis dysfunction, and others.

Regarding the type of surgery, the patients were stratified, in similarity to the STS Adult Cardiac Surgery Database, in isolated aortic valve replacement (AVR), isolated mitral valve replacement (MVR), isolated mitral valve repair (MVr), aortic valve replacement associated with coronary artery bypass surgery (AVR+CABG), mitral valve replacement associated with coronary artery bypass surgery (MVR+CABG), mitral valve repair associated with coronary artery bypass surgery (MVr+CABG), aortic valve replacement with mitral replacement or repair (AVR+MVRr), mitral valve replacement or repair associated with tricuspid valve repair (MVRr+TVr), and other operations (including pulmonary valve surgery, aortic valve replacement associated with tricuspid valve repair and isolated tricuspid valve surgery).

The European System for Cardiac Operative Risk Evaluation (EuroSCORE) I and II and surgical risk scores of all included patients were calculated based on their preoperative characteristics, surgery performed, and severity of the procedure; being used their means for analysis of the total observed mortality, type of surgery specific mortality, and etiology-specific mortality. The main data analyzed, in addition to demographic data, were those associated with the early and late surgical risks used to calculate EuroSCORE I and II. It was also included data regarding the type of surgery performed, etiology of VHD, perioperative complications, and hospital and intensive care unit (ICU) length of stay.

Postoperative complications were recorded: stroke, arrhythmia, cardiogenic shock or low-output syndrome, major bleeding, blood transfusion, acute renal failure (serum creatinine ≥ 2.0 mg/d and anuria for 12 hours or urine output < 0.3 mL/kg/h for six consecutive hours), infection, and mechanical ventilation (> 24 hours postoperatively).

### Statistical Analysis

Quantitative variables were described by mean ± standard deviation, in the presence of normal distribution, or median and interquartile range. The qualitative variables were presented by absolute frequencies (number of patients - n) and relative frequencies (percentages - %). Graphs and all analyses were performed using the statistical software R, version 3.4.3 (R Foundation for Statistical Computing).

## RESULTS

**Fig. 1:**
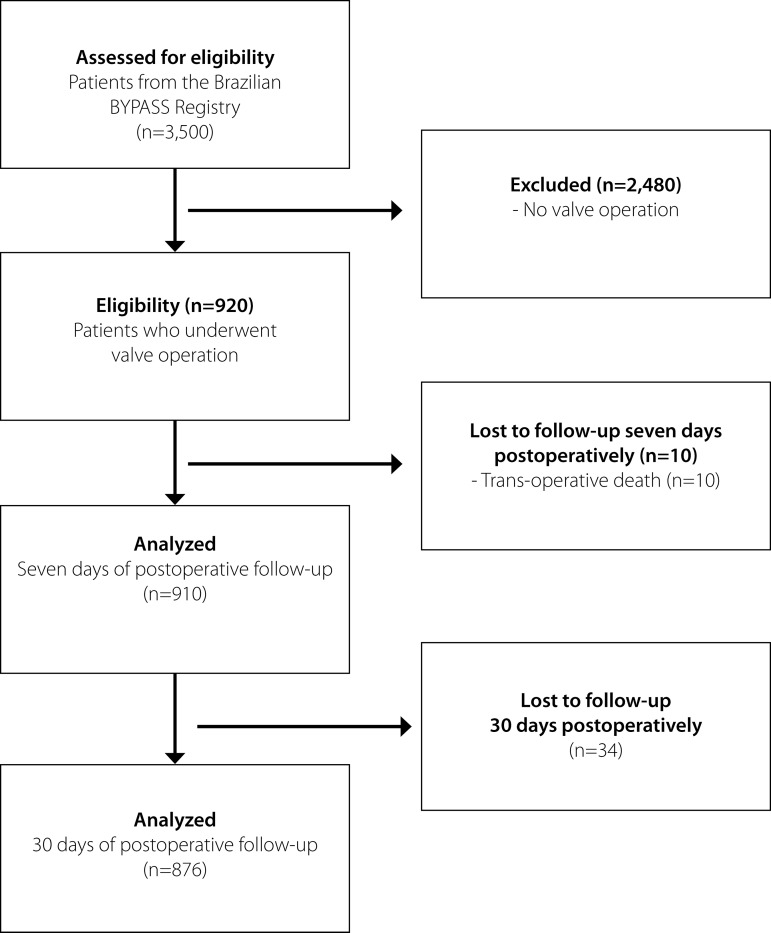
Flowchart of consecutive patients enrolled in the study. BYPASS Registry=Brazilian Registry of Cardiovascular Surgeries in Adults.

From the BYPASS study, 3,500 patients were enrolled by the participating centers between August 2014 and April 2018. In the present study encompassing valve procedures, we analyzed 920 patients in the immediate postoperative period, 910 completed follow-ups until the seventh postoperative day, and 876 patients with 30 days of follow-up, according to the flowchart (Figure 1).

The mean age of the patients was 56.7 years and women represented 47% of them. Thirty-eight percent of the patients presented with congestive heart failure, and 6.9% had left ventricular ejection fraction < 40%; 5.5% reported previous stroke and 19.9% had prior cardiac surgery. Arrhythmias were present in 22.5%, chronic obstructive pulmonary disease in 7.1%, and chronic renal failure in 5.3% of the patients included. Operations covered by the Sistema Único de Saúde (SUS), the Brazilian National Health System, accounted for 80.8%, while 19.2% were paid by the private system. The preoperative demographic and clinical characteristics of patients are listed in Table 1.

**Table 1:** Preoperative demographic and clinical characteristics.

Variables	Total (n=920)
Age (years), mean±SD	56.7±15.8
Sex (women), (n) %	(432) 47%
Type of health service system, (n) %	
The Brazilian National Health System (SUS)	744 (80.8%)
Private	176 (19.2%)
Preoperative comorbidity, (n) %	
Coronary artery disease, (n) %	186 (20.2%)
Diabetes mellitus, (n) %	159 (17.3%)
Dyslipidemia, (n) %	243 (26.4%)
Hypertension, (n) %	566 (61.5%)
Previous myocardial infarction, (n) %	52 (5.7%)
Previous cardiovascular surgery, (n) %	183 (19.9%)
Previous stroke, (n) %	51 (5.5%)
Peripheral artery disease, (n) %	(37) 4%
Congestive heart failure, (n) %	(337) 36.6%
NYHA I	(17) 5%
NYHA II	(142) 42.1%
NYHA III	(134) 39.8%
NYHA IV	(44) 13.1%
LVEF < 40%, (n) %	(58) 6.3%
Chronic renal failure, (n) %	49 (5.3%)
Dialytic, (n) %	15 (1.6%)
Current smoking, (n) %	(78) 8.5%
COPD, (n) %	(96) 7.1%
Arrhythmia, (n) %	(207) 22.5%
Active endocarditis, (n) %	(56) 6.1%
EuroSCORE I – Log, mean ± SD	7.9±10.4
EuroSCORE II – Log, mean ± SD	3.0±4.4

Data are shown as mean ± standard deviation (SD). COPD=chronic obstructive pulmonary disease; EuroSCORE=European System for Cardiac Operative Risk Evaluation; LVEF=left ventricular ejection fraction; NYHA=New York Heart Association; SUS=Sistema Único de Saúde

**Fig. 2:**
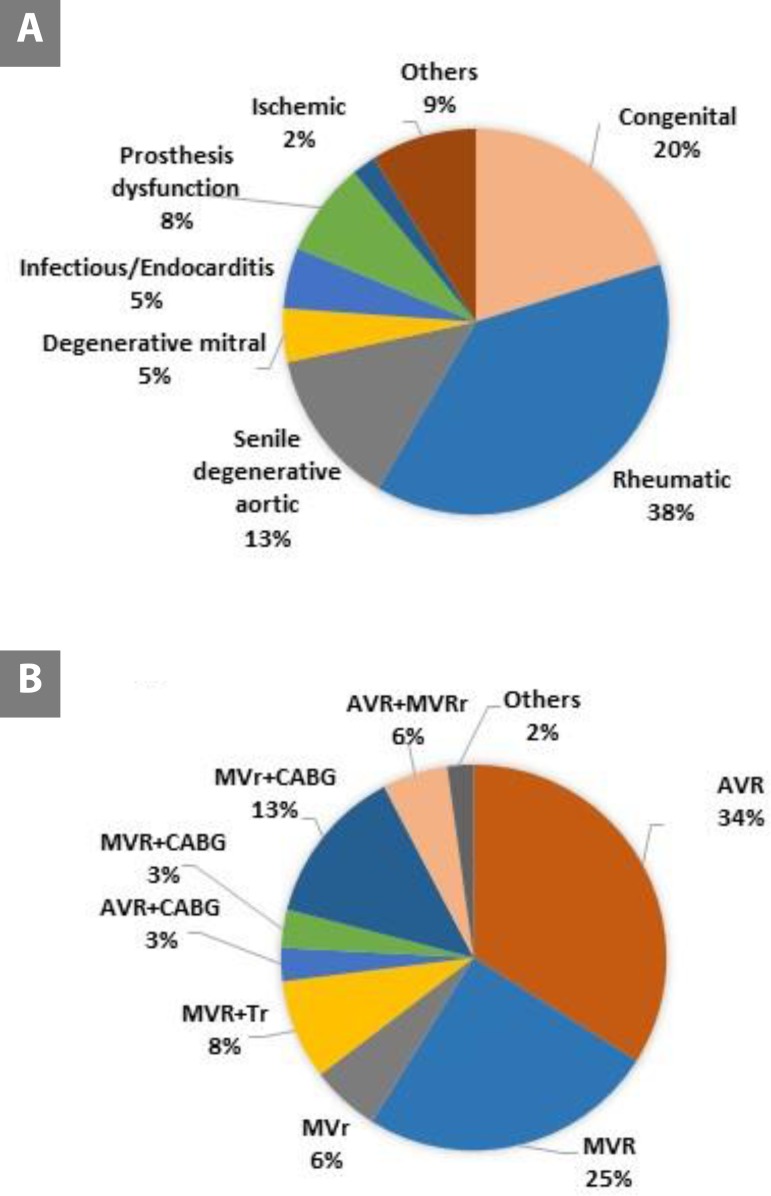
Relative frequency distribution of (A) ethiopathogeny and (B) cardiac operations by procedure type. AVR=aortic valve replacement; AVR+CABG=aortic valve replacement associated with coronary artery bypass surgery; AVR+MVRr=aortic valve replacement with mitral valve replacement or repair; MVr=mitral valve repair; MVr+CABG=mitral valve repair associated with coronary artery bypass surgery; MVR=mitral valve replacement; MVR+CABG=mitral valve replacement associated with coronary artery bypass surgery; MVRr+TVr=mitral valve replacement or repair associated with tricuspid valve repair; Others=other surgeries (including pulmonary valve surgery, aortic valve replacement associated with tricuspid valve repair and isolated tricuspid valve surgery).

Relative frequency distribution of ethiopathogeny and cardiac operations by procedure type is shown in Figure 2A. We observed RVD as the most prevalent cause of indication for surgical valve procedures in Brazil, followed by congenital etiology, senile degenerative aortic disease, degenerative mitral/leaflet prolapse or chordae rupture, and infectious/endocarditis. Ischemic etiology (functional) corresponds to only 2% of the cases.

Isolated AVR is the most frequent valve surgery performed in Brazil, followed by isolated MVR and combined MVR-AVR, as shown in Figure 2B. The distribution of the surgical access performed and trans-operative complications are seen in Table 2.

**Table 2:** Distribution of the surgical access performed and trans-operative complications.

Variables	N=920
Surgical access (n) %	
Conventional open surgery	(904) 98.3
Minimally invasive	(15)1.6
Robotic	(1) 0.1
Trans-operative complications (n) %	
Major bleeding	(91) 9.9
Blood products transfusion	(260) 28.3
Post-perfusion syndrome	(10) 1.1
Arrhythmia	(64) 7
Myocardial infarction	(2) 0.2
Low-output syndrome	(51) 5.5
Use of vasopressors	(480) 52.2
Trans-operative death	(10) 1.1

Outcomes for the more commonly performed cardiac operations are presented in Table 3. Postoperative complications occurred more frequently in combined operations, especially reoperation, like low-output syndrome, transfusion, prolonged intubation, and surgical site infection.

**Table 3:** Postoperative complications and length of stay by surgery type and total.

	AVR (n=310)	MVR (n=227)	MVr (n=53)	MVRr+ TVr (n=51)	AVR + CABG (n=77)	MVR + CABG (n=26)	MVr + CABG (n=28)	AVR + MVRr (n=121)	Others (n=20)	TOTAL (n=910)
Reoperation, (n) %	12 (3.9%)	3 (1.3%)	1 (1.9%)	0 (0%)	2 (2.7%)	0 (0%)	2 (7.1%)	5 (4.1%)	0 (0%)	25 (2.7%)
Major bleeding, (n) %	15 (4.8%)	11 (4.8%)	1 (1.9%)	2 (3.9%)	1 (1.4%)	0 (0%)	0 (0%)	8 (6.6%)	2 (10%)	40 (4.4%)
Mechanical ventilation (> 24 hours postoperatively), (n) %	18 (5.8%)	15 (6.6%)	2 (3.8%)	7 (13.7%)	12 (16.2%)	5 (19.2%)	1 (3.6%)	13 (10.7%)	2 (10%)	75 (8.2%)
Low-output syndrome, (n) %	13 (4.2%)	5 (2.2%)	0 (0%)	2 (3.9%)	8 (10.8%)	3 (11.5%)	2 (7.1%)	12 (9.9%)	1 (5%)	46 (5.1%)
Acute renal failure, (n) %	13 (4.2%)	6 (2.6%)	2 (3.8%)	3 (5.9%)	7 (9.5%)	2 (7.7%)	2 (7.1%)	6 (5%)	1 (5%)	4 (4.6%)
Coagulopathy, (n) %	9 (2.9%)	6 (2.6%)	0 (0%)	1 (2%)	2 (2.7%)	0 (0%)	0 (0%)	3 (2.5%)	0 (0%)	21 (2.3%)
Blood products transfusion, (n) %	68 (21.9%)	47 (20.7%)	11 (20.8%)	12 (23.5%)	27 (36.5%)	7 (26.9%)	9 (32.1%)	33 (27.3%)	5 (25%)	219 (24.1%)
Stroke, (n) %	3 (1%)	2 (0.9%)	0 (0%)	1 (2%)	4 (5.4%)	0 (0%)	1 (3.6%)	3 (2.5%)	0 (0%)	14 (1.5%)
Cardiac tamponade, (n) %	0 (0%)	2 (0.9%)	0 (0%)	0 (0%)	0 (0%)	0 (0%)	0 (0%)	0 (0%)	0 (0%)	2 (0.2%)
Acute myocardial infarction, (n) %	2 (0.6%)	1 (0.4%)	0 (0%)	0 (0%)	1 (1.4%)	1 (3.8%)	1 (3.6%)	0 (0%)	0 (0%)	6 (0.7%)
Vasoplegic syndrome, (n) %	3 (1%)	0 (0%)	0 (0%)	1 (2%)	3 (4.1%)	0 (0%)	2 (7.1%)	0 (0%)	0 (0%)	9 (1%)
Postoperative length of stay (days, means), %	12.5 ± 14.4	12.1 ± 8.1	9.2 ± 5.8	21.7 ± 21.7	11.1 ± 11.1	13.6 ± 8.6	11.8 ± 8.7	16 ± 10.7	16.8 ± 13.8	13.1 ± 12.4

Data from the patients who completed seven days of postoperative follow-up. AVR=aortic valve replacement; AVR+CABG=aortic valve replacement associated with coronary artery bypass surgery; AVR+MVRr=aortic valve replacement with mitral valve replacement or repair; MVr=mitral valve repair; MVr+CABG=mitral valve repair associated with coronary artery bypass surgery; MVR=mitral valve replacement; MVR+CABG=mitral valve replacement associated with coronary artery bypass surgery; MVRr+TVr=mitral valve replacement or repair associated with tricuspid valve repair; Others=other surgeries (including pulmonary valve surgery, aortic valve replacement associated with tricuspid valve repair, and isolated tricuspid valve surgery)

**Fig. 3:**
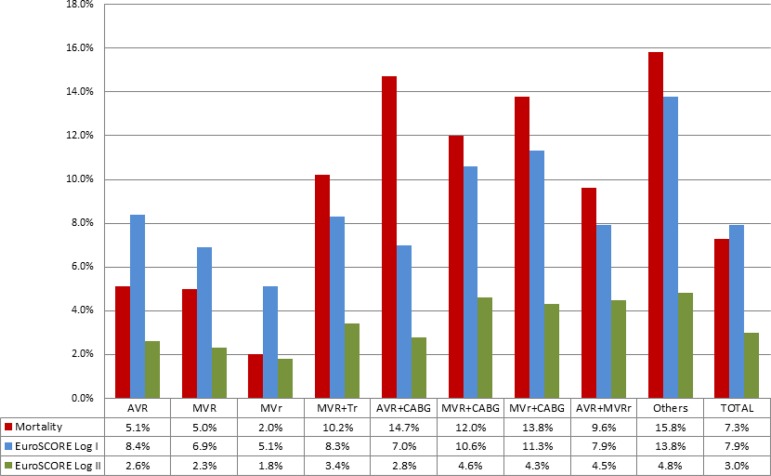
Observed mortality of patients who completed 30 days of postoperative follow-up versus predicted mortality by type of surgery. AVR=aortic valve replacement; AVR+CABG=aortic valve replacement associated with coronary artery bypass surgery; AVR+MVRr=aortic valve replacement with mitral valve replacement or repair; EuroSCORE=European System for Cardiac Operative Risk Evaluation; MVr=mitral valve repair; MVr+CABG=mitral valve repair associated with coronary artery bypass surgery; MVR=mitral valve replacement; MVR+CABG=Mitral valve replacement associated with coronary artery bypass surgery; MVRr+TVr=Mitral valve replacement or repair associated with tricuspid valve repair; Others=other surgeries (including pulmonary valve surgery, aortic valve replacement associated with tricuspid valve repair and isolated tricuspid valve surgery). Logistic EuroSCORE I and II are expressed in means by surgical type.

Mortality analysis included 876 patients who completed 30 days of postoperative follow-up. The overall mortality rate was 7.3%, while the calculated mean estimated by logistic EuroSCORE I was 7.9%. Higher operative mortality was observed in patients undergoing combined procedures (Figure 3).

**Fig. 4:**
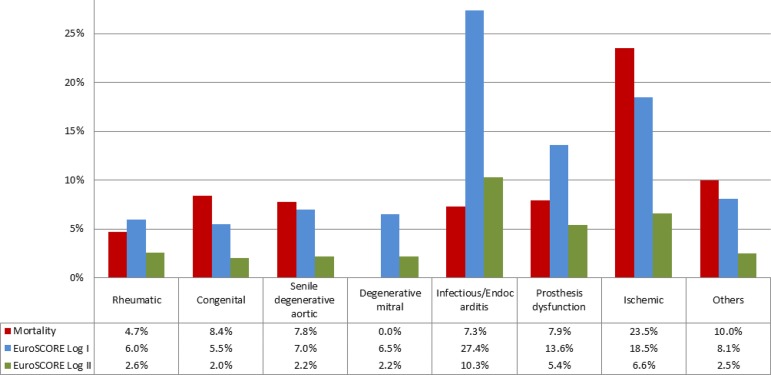
Observed mortality of patients who completed 30 days of postoperative follow-up versus predicted (EuroSCORE) due to valve disease etiology. EuroSCORE=European System for Cardiac Operative Risk Evaluation.

Figure 4 shows the mortality by valve disease etiology, compared to the predicted mortality calculated by EuroSCORE Log I and II. Table 4 compares the results of surgical mortality of valve patients from the BYPASS Registry with the STS Adult Cardiac Surgery Database 2019, as well as postoperative complications.

**Table 4:** Comparison between the Brazilian BYPASS Registry study data and the STS Adult Cardiac Surgery Database 2019 data.

	AVR		AVR + CABG		MVR		MVR + CABG		MVr		MVr + CABG	
	BYP	STS	BYP	STS	BYP	STS	BYP	STS	BYP	STS	BYP	STS
^a^Operative mortality, %	5.1	2.0	14.7	3.0	5.0	5.0	12.0	9.4	2.0	1.2	13.8	5.3
Reoperation, %	3.9	4.6	2.7	6.8	1.3	8.8	0.0	11.7	1.9	4.2	7.1	7.4
Stroke, %	1.0	1.3	5.4	1.9	0.9	2.3	0.0	0.6	0.0	1.1	3.6	3.0
Mechanical ventilation (> 24 hours postoperatively), %	5.8	6.4	16.2	11.8	6.6	17.7	19.2	28.3	3.8	5.7	3.6	19.8
Acute renal failure, %	4.2	1.8	9.5	3.8	2.6	4.9	7.7	10.3	3.8	1.7	7.1	6.0
Surgical site infection, %	0.3	0.2	4.0	0.4	0.8	0.2	3.8	0.6	3.7	0.0	2.4	0.4
Postoperative length of stay (days, means), %	12.5	6.9	11.1	8.4	12.1	10.2	13.6	12.0	9.2	6.8	11.8	10.0

a Operative mortality is defined by Society of Thoracic Surgeons (STS) databases as all deaths, regardless of cause, occurring during the hospitalization in which the operation was performed, even if after 30 days.

AVR=aortic valve replacement; AVR+CABG=aortic valve replacement associated with coronary artery bypass surgery; BYP=BYPASS Registry; BYPASS Registry=Brazilian Registry of Cardiovascular Surgeries in Adults; MVr=mitral valve repair; MVr+CABG=mitral valve repair associated with coronary artery bypass surgery; MVR=mitral valve replacement; MVR+CABG=mitral valve replacement associated with coronary artery bypass surgery

## DISCUSSION

VHD are the second leading indication for cardiac surgery worldwide, as well as in Brazil, and the rise in life expectancy of the global population brings forth the challenge of increasingly dealing with degenerative aortic and mitral valve diseases, whose incidence is expected to steadily climb over the coming decades. New minimally invasive or transcatheter techniques for treatment of valve diseases require comprehensive understanding of the surgical results of conventional techniques, costs, and prevalence of valve diseases to optimize health policies and allocate public resources.

The BYPASS Registry was conceived in 2010 and reached full operation in August 2015^[4,5]^, with the aim of providing the necessary and relevant information for improvement and implementation of cardiovascular health policies. As a reference, the STS Adult Cardiac Surgery Database was established in 1989 to address the limitations of CABG mortality data published by the federal government based solely on administrative claims data; nowadays, it is one of the most mature, comprehensive, and respected clinical data registries in healthcare. Throughout three decades of growth and refinement, it is widely recognized for accurately benchmarking risk-adjusted outcomes in cardiac surgery and serves as the foundation for all quality measurement and improvement activities, voluntary public reporting, comparative effectiveness research, and has helped to inform health policy development.

In this subset analysis of the BYPASS Registry, we identified that the most frequent VHS performed in Brazil is isolated AVR, representing one-third (34%) of all valve procedures, followed by MVR (25%), and MVr (6%). However, while in the STS database the isolated MVr accounts for more than 60% of the mitral valve operations, in Brazil, it was performed in only 26% of mitral valve procedures. This figure certainly stems from the predominance of rheumatic etiology in valve disease in our country, representing 38.3% of the cases, in which it is more difficult and less reproducible to perform reparative techniques with satisfactory surgical results.

While the degenerative etiology comprises 18% of the mitral and aortic isolated procedures, the rheumatic disease is still predominant, in stark contrast with North American figures. A retrospective analysis on 23,806 consecutive patients undergoing primary mitral valve surgery, either replacement or repair, from the Cleveland Clinic Cardiovascular Information Registry from 1993 to 2016, which represents a subset of the national STS database, the majority of patients presented with degenerative disease (58%). The next most prevalent etiologies of disease were ischemic (11.7%) and rheumatic (11.9%). In analyzing sex-specific differences among the three most prevalent disease types, women had a markedly higher rate of RVD than men^[6]^.

Ribeiro et al.^[7]^, comparing population-based data on surgical procedures to assess the relative importance of causes of heart valve disease in Salvador, Brazil, found that the most common etiologies for valve dysfunction in VHS patients were rheumatic (60.3%), degenerative (15.3%), and endocarditis (4.5%). They emphasize that RVD remains the main cause for valve surgery in Salvador, which primarily affects young adults without private health insurance. In contrast, surgery due to degenerative valve disease primarily impacts the elderly with private health insurance.

In a retrospective nationwide cohort study of 78,808 consecutive patients who underwent VHS in the SUS from 2001 to 2007, valve replacement accounted for 69.1% of the procedures performed. Mitral stenosis, the most common valve injury, represented 38.9% of the total. In 94.7% of mitral stenosis patients, the etiology was RVD and in-hospital mortality was 7.6%. Of note, etiology was improperly encoded in 35.1% of patients undergoing VHS and included in the Departamento de Informática do SUS (DATASUS), the Brazilian Health Informatics Department, thus preventing this variable to be used for risk assessment^[8]^.

In developed countries, valve repair is a standard therapy for most patients with mitral regurgitation. However, the most common etiologies for valve dysfunction in low- and middle-income countries are different from those in developed countries. Accordingly, valve repair involves different levels of challenge and outcomes in these distinct real settings.

Minimally invasive techniques advanced in the developed world, being used in almost all primary isolated degenerative mitral valve surgeries at reference centers. Contemporary reports have shown that minimally invasive technics result in mortality rates similar to conventional sternotomy, but with shorter ICU stay, decreased incidence of blood transfusion and atrial fibrillation, as well as faster post-discharge rehabilitation and recovery^[9,10]^. However, our data demonstrate that in Brazilian reality, minimally invasive access was performed in only 1.6% of the cases.

The overall mortality rate in Brazilian VHS, as unveiled is this analysis, was 7.3%, and although it was higher than that reported by the STS and the European Association of Cardiothoracic Surgery (EACTS), it is consistent with the predicted mortality calculated by the EuroSCORE I (7.9%); probably reflecting the different profile of patients operated in our country. Such evidence is repeated in analysis of mortality of subtypes of procedures, with greater mortality rates in patients with higher EuroSCORE, notably in combined surgeries (multiple valves or valve associated with CABG).

The mean age of the VHD patient referred for surgery in Brazil was 56.7 years, younger than their counterparts included in the STS database, where MVR patients had a mean of 62 years. In aortic patients, in a contemporary assessment of patients underwent surgical aortic valve replacement (SAVR) and transcatheter aortic valve replacement (TAVR), the TAVR patients were older than SAVR patients (82 *vs*. 74 years, respectively). With the rapid expansion of TAVR, our results identify some of the specific challenges that lie ahead when considering this expansion. Data on transcatheter heart valve durability and long-term outcomes in this specific cohort of younger patients will become essential^[11,12]^.

Also, the choice between biological and mechanical prosthesis, in either aortic or mitral position, should be affected by the age of the Brazilian patients coming to VHD operation. As the use of biologic valve prostheses markedly increased, the incidence of reoperation rose accordingly, with the inherent significant risks of morbidity and mortality. Even considering the emergence of the valve-in-valve procedures, it is not without risk; the reintervention itself currently carries a 7-8% mortality risk^[13]^.

The majority of patients (80.8%) were operated through SUS, whereas 19.2% were covered by private payers. This finding is in consonance with an earlier report from the BYPASS Registry, where this current share does not reflect the present distribution of private and public patients in the Brazilian healthcare system, clearly overburdening the public sector (*i.e*., the SUS)^[14]^.

The robust gathered data by the BYPASS Registry will surely help to improve quality, provide precise overview of cardiac surgical activities and patients characteristics across the country, and serve as background to define best practices and assistance with performance metrics. As solely funded by the BSCVS, including information from the public and private healthcare systems, it constitutes an independent and trusted source of information with no commercial or financial bias, providing useful tool to the government, health policy providers, healthcare regulators and developers for patient safety, the production of research protocols, and clinical improvement in cardiovascular surgery. Collaboration with other large databases on cardiac surgery is also valuable and warranted, and should be encouraged, leading to multicenter clinical studies and strengthening the evidences available so far^[15-18]^.

### Limitations

The BYPASS Registry data collected represents the experience of selected hospitals distributed across the country which voluntarily adhered to the project, providing the information required by the dedicated questionnaire. These hospitals enrolled may not be representative of the overall country standard, and further data of a registry with an expanded number of institutions will help clarify this issue. Nonetheless, the data retrieved from these institutions reassure the quality and safety of the VHS performed in Brazil

## CONCLUSION

The most frequent isolated valve surgery in Brazil is the AVR by conventional open access and the rheumatic disease is still the main etiology for valve surgery. The BYPASS Registry has a fundamental role to provide information on the profile of patients with VHD in our country in order to delineate adequate strategies for health promotion and resource allocation for cardiac surgery.

**Table:** 

Authors' roles & responsibilities	
ACZ	Substantial contributions to the conception or design of the work; or the acquisition, analysis, or interpretation of data for the work; drafting the work or revising it critically for important intellectual content; final approval of the version to be published
SG	Substantial contributions to the conception or design of the work; or the acquisition, analysis, or interpretation of data for the work; drafting the work or revising it critically for important intellectual content; final approval of the version to be published
ISR	Substantial contributions to the conception or design of the work; or the acquisition, analysis, or interpretation of data for the work; drafting the work or revising it critically for important intellectual content; final approval of the version to be published
JAES	Substantial contributions to the acquisition of data for the work; final approval of the version to be published
OB	Substantial contributions to the acquisition of data for the work; final approval of the version to be published
RAKK	Substantial contributions to the acquisition of data for the work; final approval of the version to be published
FBJ	Substantial contributions to the acquisition of data for the work; final approval of the version to be published
ABC	Substantial contributions to the acquisition of data for the work; final approval of the version to be published
RHNS	Substantial contributions to the acquisition of data for the work; final approval of the version to be published
WJG	Substantial contributions to the conception or design of the work; or the acquisition, analysis, or interpretation of data for the work; drafting the work or revising it critically for important intellectual content; final approval of the version to be published
